# Recent advances in renal regeneration

**DOI:** 10.12688/f1000research.17127.1

**Published:** 2019-02-25

**Authors:** Sho Hasegawa, Tetsuhiro Tanaka, Masaomi Nangaku

**Affiliations:** 1 Division of Nephrology and Endocrinology, University of Tokyo Graduate School of Medicine, Tokyo, Japan

**Keywords:** renegeration, pluripotent stem cells, kidney

## Abstract

Regeneration of a functional kidney from pluripotent stem cells (PSCs) is challenging because of its complex structure. Kidneys are derived from embryonic metanephros, which are composed of three progenitor cells: nephron progenitors, ureteric bud, and stromal progenitors. Nephron progenitors and ureteric bud have been induced successfully from PSCs as a result of the understanding of their detailed developmental process through cell-lineage tracing analysis. Moreover, these induced progenitors can be used to reconstruct the three-dimensional (3D) structure of kidneys *in vitro*, including glomeruli with podocytes, renal tubules, and the branching ureters. Induction of the remaining renal progenitors (that is, stromal progenitors from PSCs and the further maturation of reconstructed kidneys) needs to be studied extensively to regenerate functional and sophisticated kidneys from PSCs. In addition to the proper induction of renal progenitors, new bioengineering methods such as decellularization and 3D bioprinting and the recent advancements in the regeneration of kidneys in other species are promising leads for regenerating the complex spatial arrangement of kidneys, including the vascular network and urinary excretion pathway in humans.

## Introduction

Establishment of human induced pluripotent stem (iPS) cells was a big step that brought us closer to a realization of organ regeneration and transplantation, which was a distant dream for many researchers ^1^. Although the advancements in renal regeneration are falling behind in terms of technology compared with regeneration of other organs because of its complex structure, the strategy to induce renal three-dimensional (3D) structures *in vitro* has recently been advanced by the detailed analysis of developmental origins of the kidney. The National Institute of Diabetes and Digestive and Kidney Diseases (Bethesda, MD, USA) is now leading a consortium called “(Re)Building a Kidney” to optimize approaches for the isolation, expansion, and differentiation of appropriate kidney cell types and the integration of these cells into complex structures that replicate human kidney function ^2^, which shows the extent of attention being given to this area of research. In this review, we summarize the recent advances and future perspectives of renal regeneration.

## Three types of renal progenitor cells

**Figure 1.**
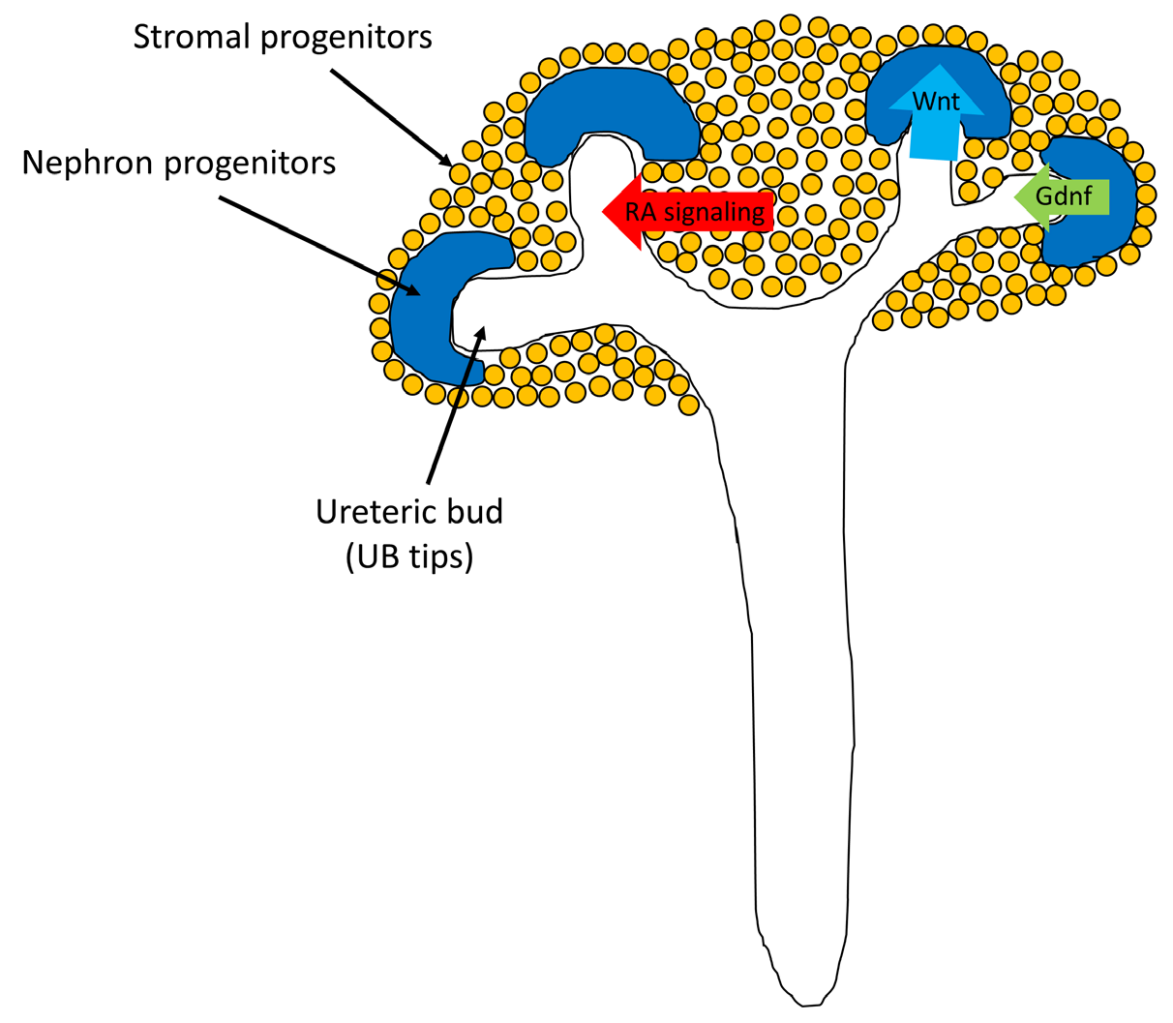
Three renal progenitors’ interaction during kidney organogenesis. Nephron progenitors, ureteric bud (UB), and stromal progenitors interact with each other to undergo kidney organogenesis. Tips of the UB (UB tips) send signals to maintain undifferentiated nephron progenitors and induce mesenchymal-to-epithelial transition of nephron progenitors by transient Wnt signaling. In turn, the undifferentiated nephron progenitors produce glial cell–derived neurotrophic factor (Gdnf) to maintain UB tip proliferation, and the stromal progenitors support ureteric branching by retinoic acid (RA) signaling.

Accurate understanding of the organogenesis process is crucial for achieving renal regeneration. Three primordia—pronephros, mesonephros, and metanephros—are developed during embryogenesis ^3^. Kidneys are derived from the embryonic metanephros, which develops at the most posterior part of the body trunk, whereas the pronephros and mesonephros develop at the more anterior part of the body trunk in earlier developmental stages, which eventually degenerate. Metanephros is formed by three progenitor cells: nephron progenitors, ureteric bud, and stromal progenitors. Nephron progenitors undergo mesenchymal-to-epithelial transition, forming glomeruli and renal tubules; ureteric bud undergoes branching morphogenesis, forming collecting ducts and ureters; and stromal progenitors differentiate into interstitial cells. Thus, the optimal induction of these three progenitor cells from pluripotent stem cells (PSCs) is a critical step in achieving renal regeneration ^4^. Moreover, the interaction between these three progenitors is important ( Figure 1). The tips of the ureteric bud (UB tips) send signals to maintain undifferentiated nephron progenitors and induce mesenchymal-to-epithelial transition of nephron progenitors by a transient Wnt signaling. In turn, the undifferentiated nephron progenitors produce glial cell–derived neurotrophic factor (Gdnf) to maintain UB tip proliferation, and the stromal progenitors support ureteric branching by retinoic acid signaling. These interactions attain nephron progenitors’ maintenance and differentiation at the same time, producing millions of nephrons with systemic connections. Therefore, the continuous supply of nephron progenitors ^5^ and ureteric branching as a result of interactions between three progenitors ^4^ are essential for organ-scale kidney morphogenesis.

## Induction of nephron progenitors from pluripotent stem cells

**Figure 2.**
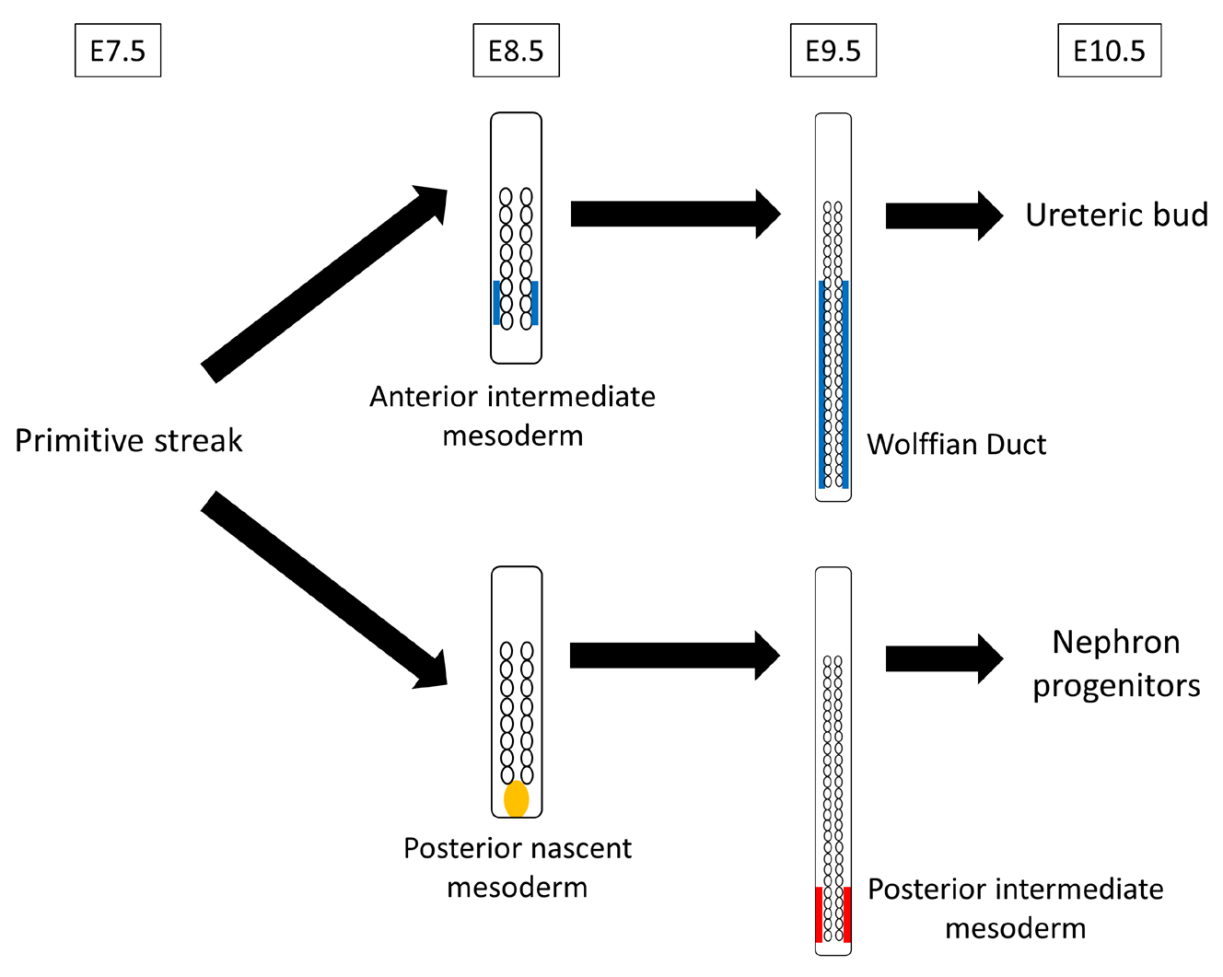
Different developmental process of nephron progenitors and ureteric bud. The precursor of ureteric bud is differentiated into anterior intermediate mesoderm on embryonic day 8.5 (E8.5) and forms the Wolffian duct on E9.5. The precursor of nephron progenitors is maintained and localized posteriorly in an undifferentiated state (posterior nascent mesoderm) until E8.5, differentiates into posterior intermediate mesoderm on E9.5, and subsequently becomes nephron progenitors.

**Figure 3.**
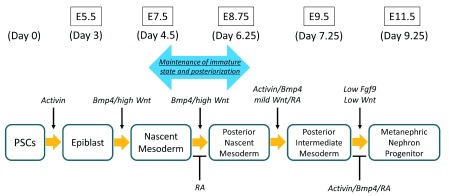
“Anterior/posterior intermediate mesoderm concept” for the accurate induction of nephron progenitors. Considering that the precursor of nephron progenitors is maintained and localized posteriorly in an undifferentiated state (posterior nascent mesoderm) until embryonic day 8.5 (E8.5), Taguchi *et al*. ^12^ used an unusually high concentration of Wnt agonist to maintain the immature state during the posteriorization phase. Subsequently, graded attenuation of the Wnt agonist, as well as stage-specific addition of growth factors, led to metanephric nephron progenitor formation. The required signaling for the lineage specification at each embryonic stage (E) and the *in vitro* differentiation timing (Day) of human induced pluripotent stem (iPS) cells are shown ^4, 12^. Bmp4, bone morphogenetic protein 4; Fgf9, fibroblast growth factor 9; RA, retinoic acid.

Both nephron progenitors and ureteric bud were believed to be derived from the same intermediate mesoderm, which appears on about embryonic day 8.5 (E8.5) and expresses paired box gene 2 ( *Pax2*) ^6, 7^. Although the intermediate mesoderm-like cells have been successfully induced from PSCs ^8– 10^, the 3D structure of kidneys could not be regenerated from it. There is a possibility that the induced cells were not intermediate mesoderm but lateral plate mesoderm, for Mae *et al*. have not investigated the possibility of lateral plate mesoderm induction in their generating cells although they used odd skipped–related 1 ( *Osr1*) as the selection marker ^8^, which was thought to be specifically expressed in the intermediate mesoderm but, in fact, is also expressed in other areas such as lateral plate mesoderm. The cell-lineage tracing analysis ^11^ recently revealed that precursor of nephron progenitors, which is T (Brachyury)-positive, is maintained and localized posteriorly in the undifferentiated state (posterior nascent mesoderm) until E8.5 and differentiates into posterior intermediate mesoderm at E9.5, which subsequently becomes nephron progenitors ( Figure 2). This, however, is against the conventional concept which states that the entire kidney is derived from early-stage intermediate mesoderm which overlaps with recently recognized anterior intermediate mesoderm. Thus, nephron progenitors are different from ureteric bud in both their origin (nephron progenitors originate from posterior/late-stage intermediate mesoderm, whereas ureteric bud originates from anterior/early-stage intermediate mesoderm) and the timing of intermediate mesoderm differentiation (nephron progenitor lineage differentiates on E9.5, and ureteric bud lineage differentiates on E8.5). Owing to this detailed understanding of the kidney development process, Taguchi *et al*. succeeded in inducing nephron progenitor cells from mouse embryonic stem (ES) cells and human iPS cells via posterior nascent mesoderm and posterior intermediate mesoderm ( Figure 3) ^12^. They succeeded in maintaining the immature mesoderm state (T-positive) during the posteriorization phase by using an unusually high concentration of Wnt agonist. Subsequently, graded attenuation of the Wnt agonist, as well as stage-specific addition of growth factors, led to metanephric nephron progenitor formation. Moreover, they demonstrated that the induced nephron progenitors reconstituted the 3D structure of kidneys *in vitro*, including glomeruli with podocytes and renal tubules with proximal and distal regions ^12^.

Based on this concept, Morizane *et al*. successfully developed a more efficient method for inducing nephron progenitor cells ^13^. Furthermore, some researchers have developed a method for selectively expanding nephron progenitors derived from mouse embryos, mouse ES cells, and human iPS cells ^5, 14, 15^, which would help to reconstruct kidneys *in vitro* because the continuous supply of nephron progenitor cells is essential.

## Induction of ureteric bud from pluripotent stem cells

Proper organization of ureteric branching morphogenesis is essential for kidney function. Takasato *et al*. generated kidney organoids by induction of multiple kidney components, including nephron progenitors, ureteric bud, and stromal progenitors, using a single protocol ^16^, but their organoids did not have a dichotomously branching ureteric tree. Taguchi and Nishinakamura induced ureteric bud from mouse ES cells and human iPS cells by using a protocol completely different from that used for the induction of nephron progenitors ^4^. Since the duration of immature mesoderm state is different between nephron progenitors and ureteric bud, they regulated it by changing the exposure time to the Wnt signaling ^4, 17^. Furthermore, preparing a budding ureteric structure with an isolated E11.5 metanephric mesenchyme (including nephron progenitors and stromal progenitors) resulted in the dichotomous branching up to six or seven generations of ureteric bud with nephron progenitors on each ureteric bud tip (nephron progenitor niches) and formed differentiated nephrons containing distal and proximal tubules along with glomerular structures, suggesting how important it is to control the spatial arrangement of progenitor cells. This higher-order kidney organoid (branching ureter with nephron progenitor niches and differentiated nephron components) could also be reconstructed by adding separately induced nephron progenitors and ureteric bud from mouse ES cells to stromal progenitors sorted from E11.5 embryonic kidneys. ****

## Future challenges for kidney regeneration *in vitro*

As mentioned above, rapid and remarkable advances have been made in the field of kidney regeneration, but reconstructing functional and sophisticated kidneys from PSCs is still a challenging task.

First, signals required for induction of the stromal progenitor lineage remain to be elucidated, even though the selective induction of the other two renal progenitors (nephron progenitors and ureteric bud) from PSCs has already been established. Kobayashi *et al*. revealed that *Foxd1*-expressing cortical stroma represents a distinct multipotent self-renewing stromal progenitor population that gives rise to stromal tissues using cell-fate mapping analysis ^18^. The detailed differentiation process of stromal progenitors, however, has not yet been clearly understood. Thus, Taguchi and Nishinakamura still used stromal progenitors sorted from mouse embryonic kidneys when reconstructing higher-order kidney structures with separately induced nephron progenitors and ureteric bud from PSCs ^4^. Since primary human embryonic stromal progenitors are not readily available, induction of stromal progenitors from PSCs is strongly required. Although Takasato *et al*. have reported that multiple kidney components, including stromal progenitors, can be induced from PSCs by a single protocol ^16^, this kidney organoid has limited functionality and no ureteric bud cell types were detectable by single-cell RNA sequencing ^19^. Given that nephron progenitors and ureteric bud have distinct origins, selective induction of the three renal progenitors from PSCs is one of the best strategies for reconstructing higher-order kidneys at this stage. Therefore, it is critical to understand the differentiation process of stromal progenitors and establish the optimal conditions for their induction from PSCs for the reconstruction of sophisticated and functional kidneys *in vitro*.

Second, at present, the reconstructed kidney organoid has not fully matured in scale, structure, and function. Although Taguchi and Nishinakamura were successful in reconstructing higher-order kidney organoid (branching ureter with nephron progenitor niches and differentiated nephron components), cDNA microarray analyses of their reconstructed kidney organoids revealed that their organoids cultured for 7 days most closely resembled the E15.5 kidney ^4^, indicating that they were different from adult kidneys. It was speculated that blood supply might be required to make the organoids function as kidneys. Takebe *et al*. showed that liver buds generated from human iPS cells developed into vascularized and functional human livers by murine intracranial or mesenteric transplantation ^20^, showing that blood perfusion is important for making reconstructed organs functional. Sharmin *et al*. transplanted human iPS cell–derived nephron progenitors beneath the kidney capsule of immunodeficient mice and demonstrated that the human glomeruli were vascularized by the host endothelial cells, resulting in further maturation of podocytes ^21^. Van den Berg *et al*. ^22^ have also shown that renal subcapsular transplantation in mice induces vascularization with blood perfusion of human iPS–derived kidney organoids reconstructed by Takasato’s protocol ^23^, resulting in progressive maturation of nephron structures such as podocyte foot processes and polarization and segmental specialization of tubular epithelium. Although the transplantation approach by Sharmin *et al*. required the addition of vascular endothelial growth factor (VEGF) to the transplant ^21^, Van den Berg *et al*. have shown that the kidney organoids themselves actively secrete VEGF and induce host-derived angiogenic vascularization after transplantation ^22^. However, the vascular system formed in the transplanted kidney organoids is simple at this stage and there is still a very long way to go before the appropriate kidney vascular networks can be reproduced.

Furthermore, using kidney organoids as disease modeling might contribute to the medical research. Cruz *et al*. have generated a genetic model of polycystic kidney disease using human iPS cells and established a highly efficient model of cystogenesis ^24^. Hale *et al*. have shown that podocytes in kidney organoids have improved podocyte-specific gene expression and polarized protein localization compared with podocyte cell lines cultured in 2D ^25^. Tanigawa *et al*. established kidney organoids using human iPS cells from a patient with an NPHS1 missense mutation, identifying impaired nephrin localization and slit diaphragm formation in podocytes ^26^. In this context, how to standardize the differentiation efficiency among the PSCs which have a different genetic background is a significant challenge. Czerniecki *et al*. recently presented a protocol for the miniaturization and automation of human organoid differentiation from iPS cells, showing that kidney organoids can be applied to high-throughput screening focusing on therapeutic discovery and toxicology ^27^.

## Future perspectives for regenerating functional kidney *in vivo*

Application of recent technological advancements in human regenerative medicine can help in regenerating complex spatial arrangement of kidneys with vascular network and urinary excretion pathway.

One promising strategy might be a bioengineering technique such as decellularization and 3D bioprinting. Song *et al*. decellularized rat kidneys by detergent perfusion, which yielded acellular scaffolds, and then seeded them with epithelial and endothelial cells and perfused these cell-seeded constructs in a whole-organ bioreactor ^28^. Although transplantation of this bioengineered kidney exhibited excretory function *in vivo*, optimization of cell-seeding protocols and upscaling of biomimetic organ culture are still required for their use in clinical settings. Application of 3D bioprinting methods has also been successful in reconstructing the complicated structures of proximal tubules ^29^ and vasculatures ^30, 31^ *in vitro*, although the physiological functions reproduced by these technologies reflect only a small part of organs.

Another promising strategy might be regenerating human kidneys in other species. Kobayashi *et al*. first generated rat pancreas in mouse via interspecific blastocyst complementation ^32^. Yamanaka *et al*. applied this concept and succeeded in regenerating rat-derived nephrons in mice by combining the transplantation of rat-derived nephron progenitors with the native nephron progenitors’ conditional elimination ^33^, thereby demonstrating a technical platform for regenerating kidneys in other species. The same group was successful in demonstrating a stepwise peristaltic ureter system for constructing the urinary excretion pathway in stem cell–generated embryonic kidneys ^34^. Concretely, rat metanephroi with bladders developed from cloacas were transplanted into host rats and then were connected to the host animal’s ureter (a stepwise peristaltic ureter system). Thus, functional kidneys can be theoretically reconstructed by the combination of three technologies—induction of nephron progenitors from human iPS cells, regeneration of human nephrons in other species, and construction of a urinary excretion pathway—which might be the most promising strategy for regenerative medicine at present. However, there is still a big ethical problem regarding the generation of chimeric animals.

## Conclusions

Although regeneration of a functional kidney is difficult because of its complex structure, recent advancements in this field are remarkable. The cell-lineage tracing analysis has revealed details of the developmental process of renal progenitors, which allows the induction of two of the three renal progenitor cells from PSCs and the reconstruction of higher-order kidney organoids *in vitro*, even though the degree of maturation of these organoids is not satisfactory. Combining the induction of renal progenitors from PSCs with new bioengineering methods, including decellularization and 3D bioprinting, and the recent advancements in the regeneration of kidneys in other species would be a promising strategy for regenerating functional human kidneys.
